#  “Eating Out”, spatiality, temporality and sociality. A database for China, Indonesia, Japan, Malaysia, Singapore and France

**DOI:** 10.3389/fnut.2023.1066737

**Published:** 2023-02-02

**Authors:** Elise Mognard, Kremlasen Naidoo, Cyrille Laporte, Laurence Tibère, Yasmine Alem, Helda Khusun, Judhiastuty Februhartanty, Yoko Niiyama, Haruka Ueda, Anindita Dasgupta, Anne Dupuy, Amandine Rochedy, Jan Li Yuen, Mohd Noor Ismail, Pradeep Kumar Nair, Neethianhantan Ari Ragavan, Jean-Pierre Poulain

**Affiliations:** ^1^Faculty of Social Sciences and Leisure Management, Taylor's University, Subang Jaya, Malaysia; ^2^“Food Studies: Food, Cultures and Health”, Université de Toulouse, Toulouse, France; ^3^Center for Asian Modernisation Studies (CAMS), Taylor's University, Subang Jaya, Malaysia; ^4^Centre d'Études et de Recherche: Travail, Organisation, Pouvoir (CERTOP) Unité Mixte de Recherche 5044 (UMR), Centre National de la Recherche Scientifique (CNRS), Université de Toulouse, Toulouse, France; ^5^SEAMEO Regional Center for Food and Nutrition (RECFON) – Pusat Kajian Gizi Regional Universitas Indonesia, Jakarta, Indonesia; ^6^College of Gastronomy and Management and Graduate School of Economics, Ritsumeikan University, Kyoto, Japan; ^7^Japan Society for the Promotion of Sciences, Tokyo, Japan; ^8^Graduate School of Environmental Studies, Nagoya University, Nagoya, Japan; ^9^Centre for Community Health Studies (ReaCH), Faculty of Health Sciences, Universiti Kebangsaan Malaysia, Bandar Baru Bangi, Malaysia

**Keywords:** sociology of food, health, nutrition, eating out, food practices, food transition, compacted modernization, open science

## 1. Introduction

Eating out is a central dimension of the food and nutrition transitions (1–3). However, most of the available data on eating out were reported in Europe and North America. A high rate of eating out is one of the specificities of the Asian food system (4–6), which is further assumed to increase alongside compressed modernization (7–9). To fill this gap, “Eating Out” is a recurrent cross-sectional survey that focuses on the spatiality, temporality, and sociality of food intakes in five Asian countries and one European country. It, thus, addresses an important data gap by allowing cross-national comparisons and quantitative assessments of movements of food between home and out of home across a large consortium. It is conducted within the framework of the chair of “Food Studies: Food, Cultures, and Health” created jointly by Taylor's University (Malaysia) and the University of Toulouse Jean Jaurès (France), in partnership with SEAMEO RECFON (Indonesia) and Ritsumeikan University (Japan), and lead by Jean-Pierre Poulain. This survey is a part of the wider Asian Food Barometer initiative and supplementary to the national Food Barometers, currently, the Malaysian (10) and the Indonesian databases. While the national surveys are including data on the food content and quantities, thus enabling analysis of the nutrient composition (11), “Eating Out” is focusing on the food day patterns.

This article briefly reviews the available data on eating out—specifically in Asia, proposes a framework, and details the methods regarding the organization of the initial data collection (2019–2020). Expected uses and limitations of the data as well as their possible contributions conclude the article.

## 2. Empirical data on eating out

While most agree that the prevalence of eating out in Asia is high, its analysis mainly relies on economic and nutritional data that focus, respectively, on monetary flows and nutritional intakes. In addition, the empirical analysis of the behavioral dimension is faint.^1^ In addition, one of the challenges of an empirical study of eating out practices mainly lies in the polysemy of “consumption”, where diverse behaviors are possibly aggregated on the same site: purchases and actual individual incorporation, which are framed by different—and at times conflicting—angles (12–14).

For the purchases, data collected by the household consumption and expenditure surveys primarily aim at deriving consumption patterns and providing input to the compilation of national accounts, from the economic perspective. Those surveys refer to the food prepared away from home, either purchased—from a commercial establishment, a canteen or cafeteria at a school or at the workplace—or received in-kind from a school or an employer, a food assistance program, or a gift from another household (15). Conversely, they typically exclude or aggregate in other expenditure food intakes within institutional care or business meals. Additional insights are provided by specialized consultants in the food service industry and generalist global market research companies (16) with the relationships between individual preferences and socio-economic variables. However, the limited access to the methodology—due to the cost incurred—constrains the identification of the objectivity of the data collected, which can vary from the declared practices to social representations.

Regarding the actual individual incorporation, numerous nutritional studies reflect the public health concern with eating out. They contribute to the analysis of individual diets in terms of nutritional composition—that is assumed to be higher in energy, fat, sugar, and salt and low in vegetables—which happens to be the reverse of dietary advice. Nonetheless, the heterogeneity in the definitions of eating out (17–19) makes difficult cross-national comparisons or attempts to quantitatively assess a change (20). Most importantly, they do not reflect movements of food between home and out of home. Globally, social scientists are interested in “Eating Out” from what it says about the character of contemporary societies and provides details on the diverse socio-cultural and socio-historical meanings of eating out and focuses on the socio-technical and institutional arrangements (21–30). However, the empirical knowledge about food habits focuses mainly on the domestic dimension (5) and concentrates mainly on Europe and America.

**Figure 1:**
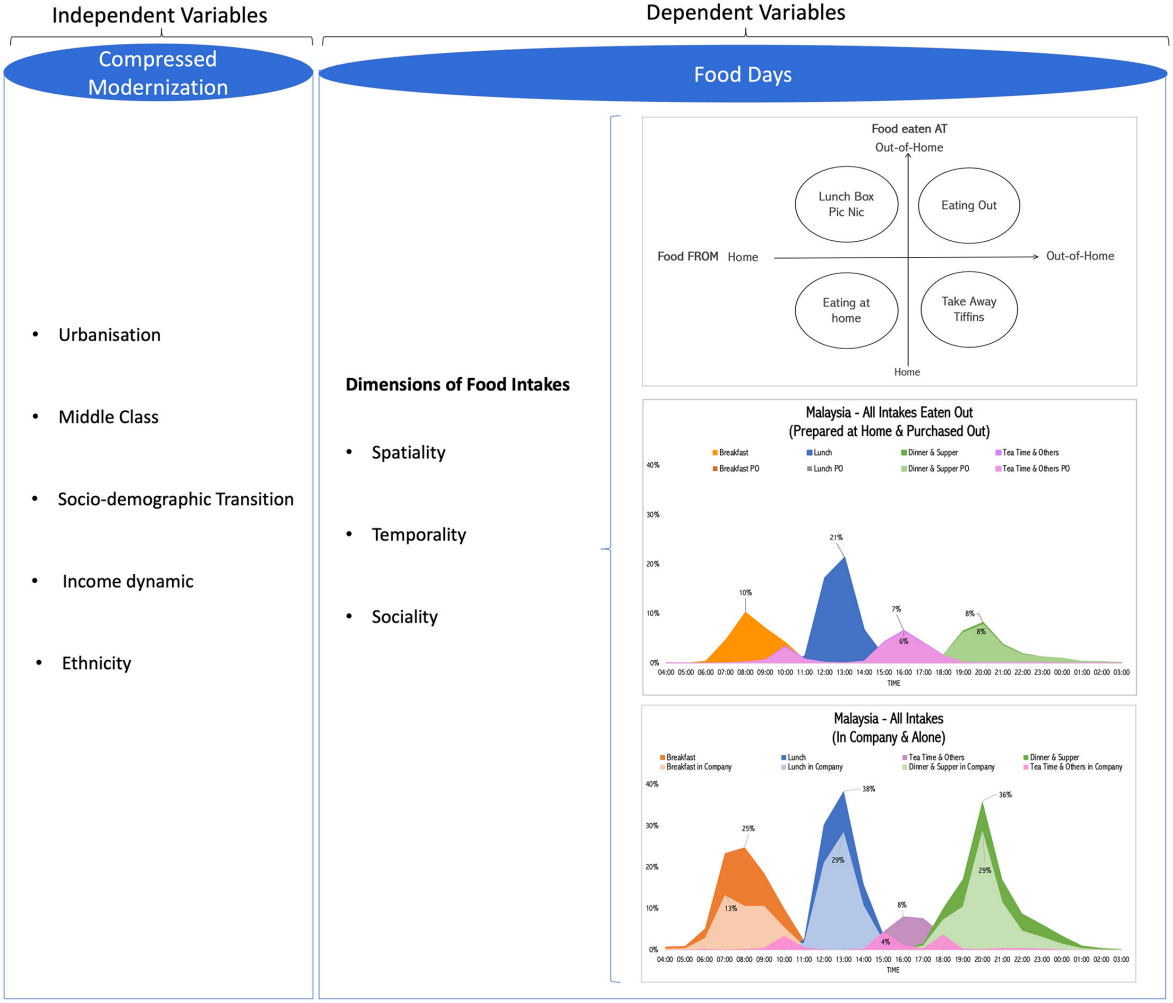
Research framework of “Eating Out” initiative.

“Eating Out” initiative posits that food decisions are embedded within behavioral scripts, routines, or rules predefined by socio-cultural contexts (31–34). These scripts, routines, or rules allow the coordination of the social actors involved in the production, processing, distribution, preparation, and incorporation of food. Therefore, they are contributing to the synchronization (13, 35, 36) and “orchestration” (23, 33, 37) of the food practices. The food habits at home interplay with the structure of the household, the number of diners, and gender roles—to name a few. When eating out, the place, individualization of the items, relation between the client, consumer, and service provider, and policies to support eating at school or workplace—among others, are essential in the definition of food social norms and practices (38, 39). Thus, the “Eating Out” initiative considers the transformations of the societies along with compressed modernization^2^ and its consequences on “food days”^3^ (12, 40). It is focused on the spatiality of preparation and eating, the temporality, and the sociality of food intake. Figure 1 summarizes the research framework.

In Asia, it seems that the role of home-cooked food in practices is not as central^4^ while the prevalence of eating out is high. Paradoxically, empirical social scientific studies at the national or cross-national levels are scarce, as mentioned earlier. The development of important economic factors in the food service sector in the 1970s in Europe (38, 39) has contributed to the production of data that have made this phenomenon visible. When existing, studies are mainly framed by the nutritional perspective [for example (42–46)] which, while being a matter of controversies in the West (47–49), is further applied without much consideration paid to the particularities of the Asian contexts and histories. Thus, it has been undertaken by the Asian Food Barometer initiative (6, 10, 50). “Eating Out” database contributes with a focus on the movements of food intake between home and out of home in relation to compressed modernization and provides an empirical basis to the debate of the social and public health implications of eating out in the Asian contexts. Four food spaces are identified to describe the movements of food between home and out of home: (1) home food—food prepared at home can include the use of convenience products; (2) eating out—food is consumed at an out-of-home outlet/restaurant/stall/canteen, etc., or on the go; (3) delivery/takeaway—food prepared out of home and consumed within the home; and (4) food prepared at home and eaten out of home.

## 3. Methods

### 3.1. Sample size and methods

**Figure 2:**
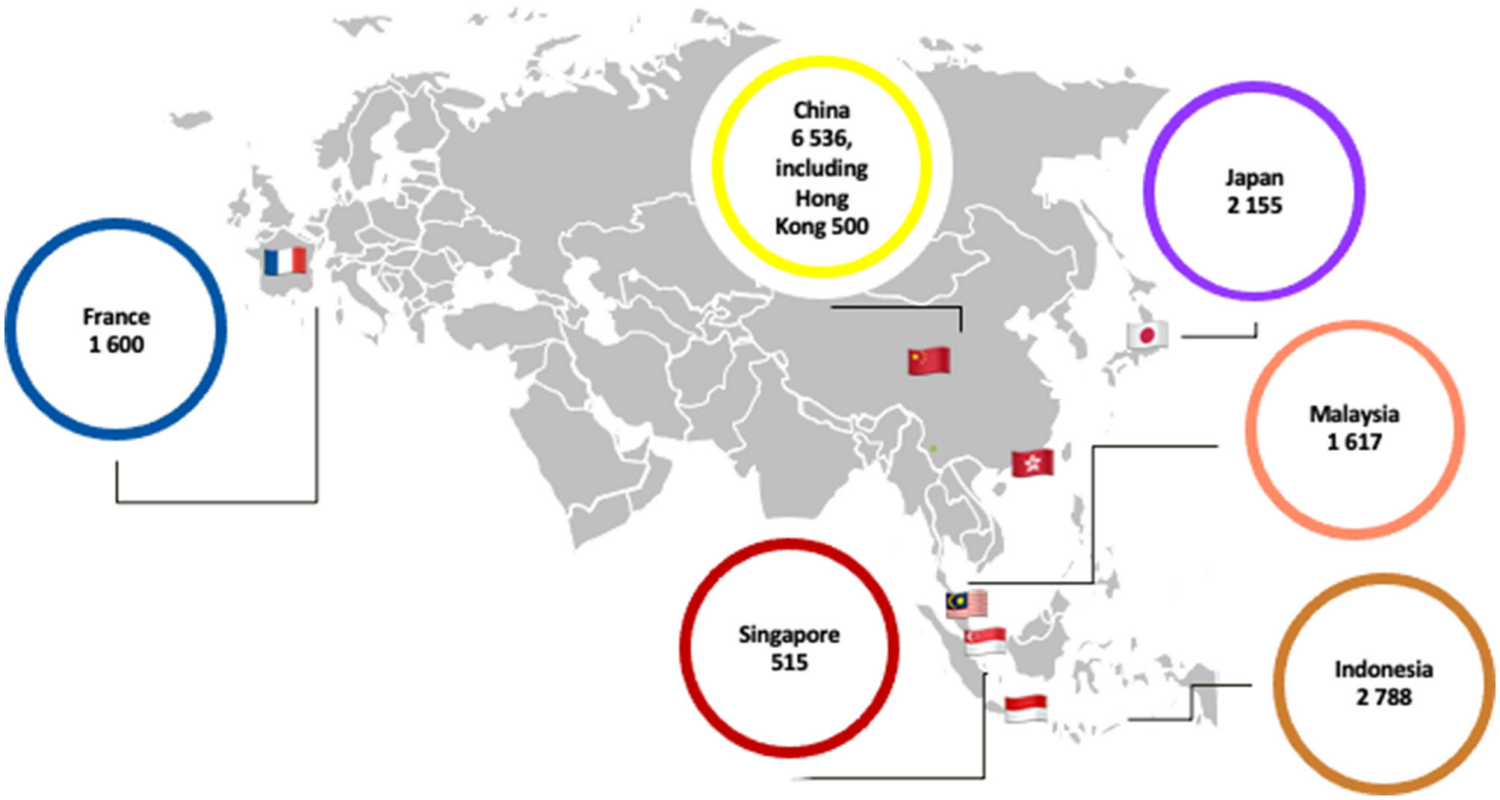
Sample of initial “Eating Out” data collection (*N* = 15,211).

The total sample size is over 15,000 respondents, following the geographical repartition presented in Figure 2. Locations chosen for the initial data collection of “Eating Out” were concentrated on East and Southeast Asia—with France providing an occidental comparison point—based on their geographical positions, population, and modernization dynamics, reported rates of eating out, shared and divergent histories, and public health concerns.

A combination of stratified random sampling—selection from panel respondent sources—and quota sampling was applied to optimize the benefits of both sampling methods. The data collection aimed at achieving national representative samples. For China, global representativity is practically very difficult. The sample focuses on a range of medium urbanized areas. Minimum sample sizes were set for each location. Respondents were selected out of the national populations aged 18 years and older, across all income groups and rural and urban areas. Quotas were implemented in each location, so to minimize challenges in representativeness for the samples on age, gender, urbanization (except for metropolitan areas of Hong Kong and Singapore), and ethnicity where relevant (Singapore and Malaysia).

The inclusion of France in this survey is first justified by the frequency of eating out in Europe^5^ that positions France as a European representative where little variations are observed compared with Asia. Second, the involvement of the research team in investing and analyzing eating out in France for approximately 30 years along with six national surveys among which one was completed with INPES (12, 13, 52). This long-term involvement constitutes both a methodological heritage and a possibility of a critical discussion with the longitudinal analysis previously developed in French data.

### 3.2. Research instrument

A structured self-administered online survey was deemed as an appropriate method to both collect data and manage the constraints of geography, linguistics, time, and budget. Given that eating out practices may differ across the week, the survey collects data based on a 72-h recall, a measure of food intake covering typically 1 to 3 days and initially developed by Wiehl (53).

**Figure 3:**
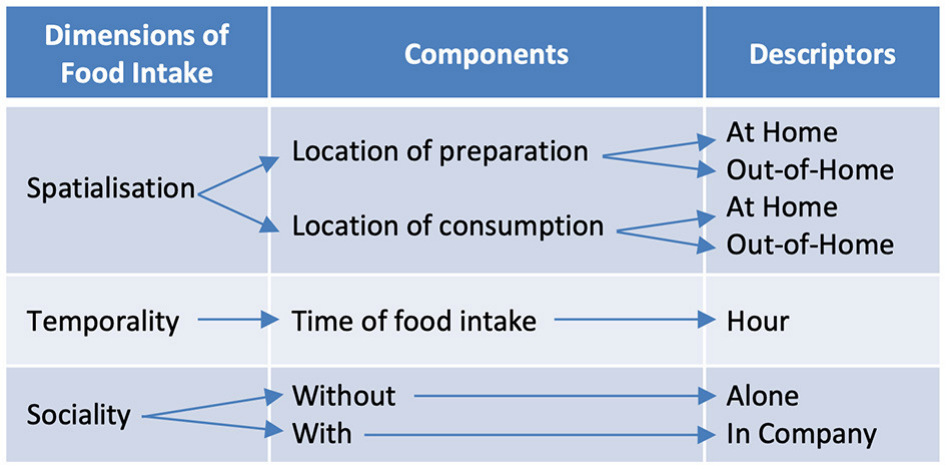
Dimensions, components, and descriptors of food intakes.

The close-ended questionnaire assisted the respondents to recall their food intakes according to their (Figure 3):

- Temporality, the recall of 3 days with the time of the food intakes was supported by a matrix breaking down each of their days into hours, starting early morning–4 a.m.–until late night–3 a.m.;
- Formality, selection of the name of the intake from a list comprising “Breakfast”, “Teatime”, “Lunch”, “Dinner”, and “Supper” and the possibility to define it as “Others”;
- Spatiality, from two options, namely, “At home” vs. “Out of the Home” and source of the food from “Food prepared at home” vs. “Food Purchased outside or delivery”;
- Sociality, between “Alone” vs. “With company”.

Invitations to participate in the survey were staged in the week and sent out on Tuesdays with the aim to spread out 3-day recall across 2 weeks to include at least 1 day from the weekend (defined as Saturday and Sunday).

The country of the collection was automatically detected when the respondent started the questionnaire. First, questions were to filter the respondents based on quotas and collect data on age, race/ethnicity, religion, gender, number of children, number of family members living under the same roof, urbanization, education attainment, monthly household income, or Wealth Index for Indonesia. Given the potential interest of the database for public health, height and weight were also included to allow the computation of the body mass index (BMI) as an indicator of nutritional status.

The questionnaire was initially designed in English. Questions were translated into the national language(s) where needed. In that case, back-translation was performed to ensure the accuracy of the translation.^6^ Typically, a respondent needed approximately 12 min to answer the questionnaire.

### 3.3. Data collection and procedures

The data for the initial survey were collected in partnership with a company—*Toluna*, specializing in conducting large multilocation surveys and holding large panels and affiliated networks in each of the locations of the “Eating Out” data collection. Invitations to participate were sent using email, mobile text, or on the partner company's mobile application platforms.^7^ The data were collected in two windows-−6 January to 24 January 2020 and 31 January to 9 February 2020.^8^

The research team has developed along with *Toluna*, a web interface, to collect data in diverse languages and unit systems. The design of the 3-day recall presents a break of each of their days into hours, starting early morning–4 a.m.–until late night–3 a.m. Any time the respondent selected a type of intake for a given time, the selection of locations of food preparation, and consumption as well as the sociality of the intake was made available.

### 3.4. Illustration of outcomes

**Figure 4:**
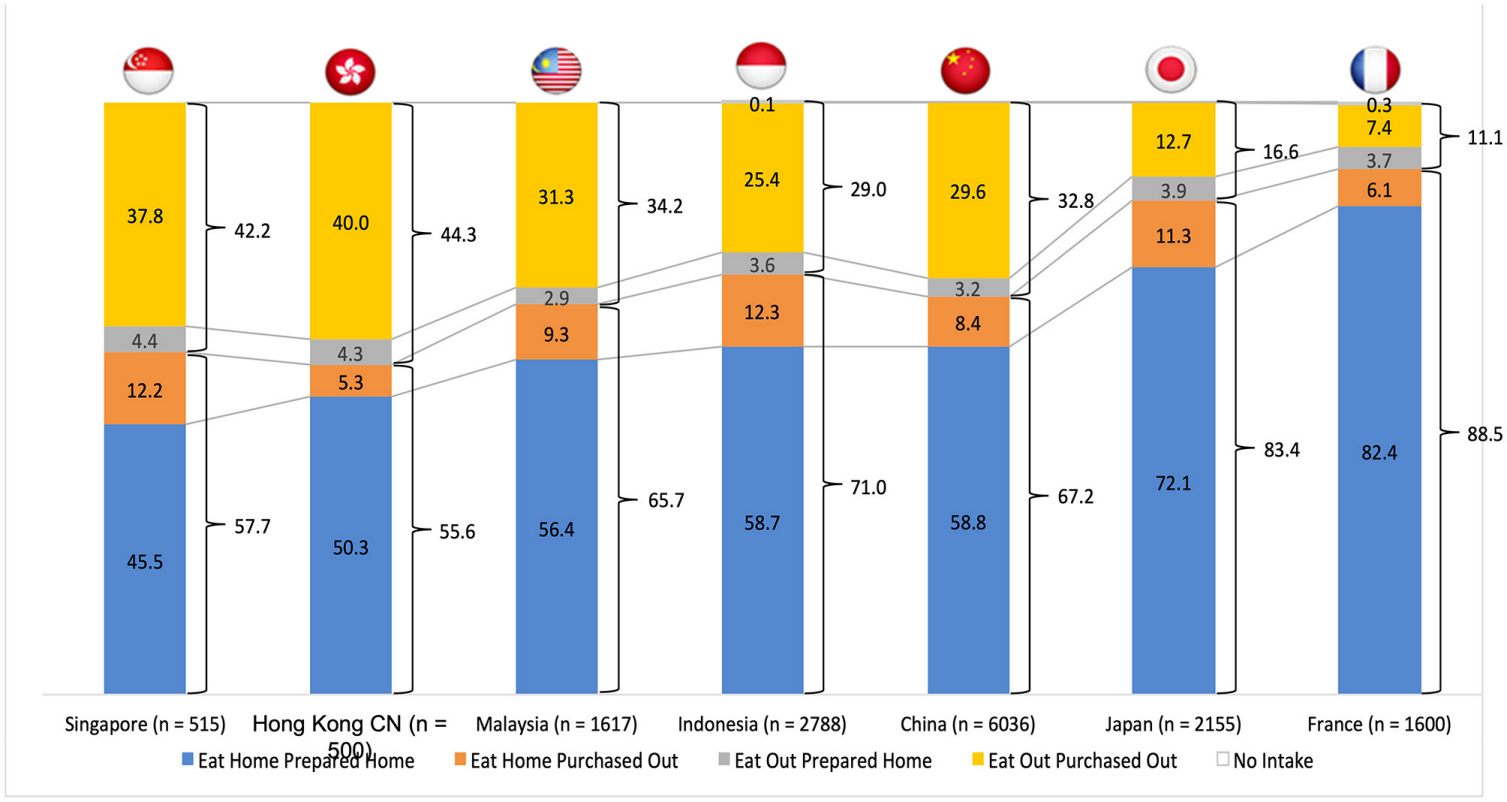
Comparison of the percentages of spatiality for preparation and consumption of meals.

The dataset provided allows analysis and comparisons of the food days across the countries based on the computation of the distribution of meals or food intakes according to the spatiality of their preparation and incorporation and their sociality as well as their temporal distribution across the time of the day. A usual statistical approach to the analysis of the variance can be applied. Figure 4 illustrates the important contrast regarding the distribution of the spatiality of food between Singapore and France—where the percentages of meals purchased out of home and either consumed at home or out of home are 50 and 13.5%, respectively.

**Figure 5:**
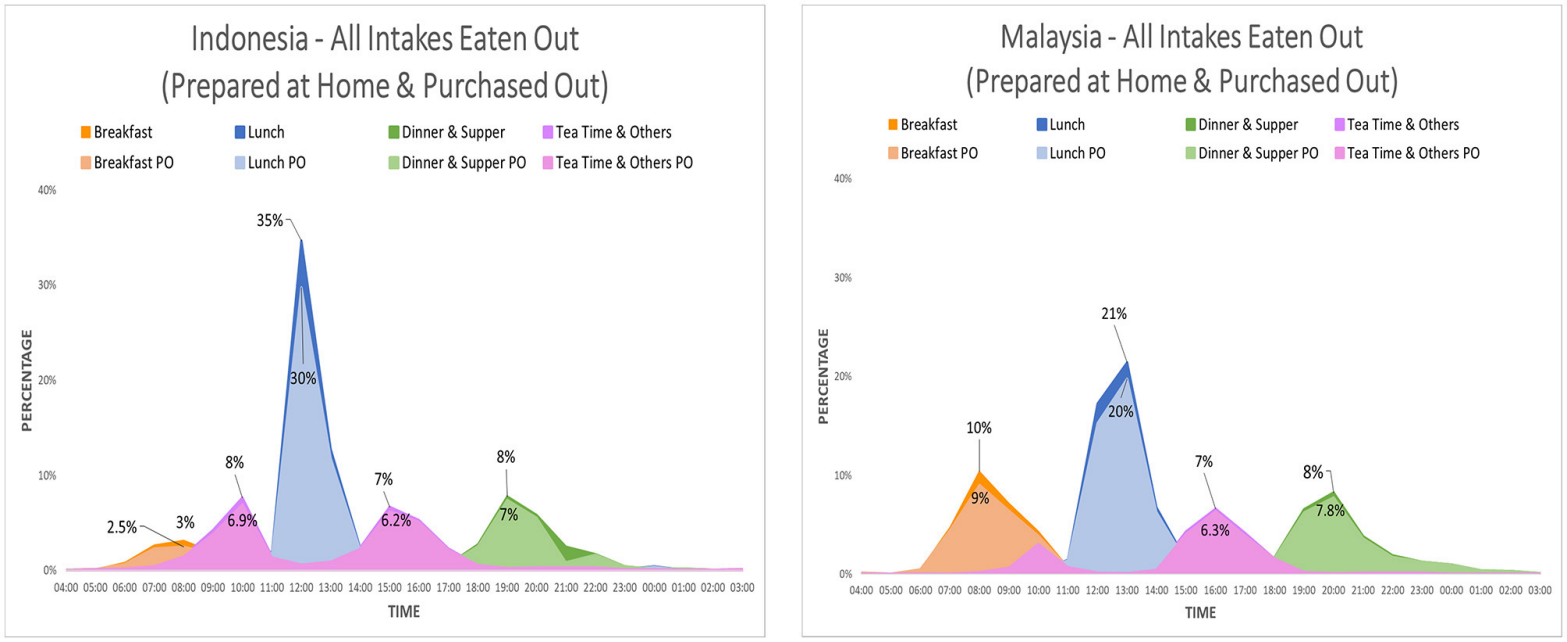
Temporal distribution of food intakes according to spatiality in Indonesia and Malaysia.

The comparison between the temporal distribution of food intakes eaten out in Indonesia and Malaysia presented in Figure 5 shows the difference in terms of the percentage of breakfast eaten out or the synchronization of the lunch eaten out where 35% of the Indonesian population eaten out at noon while 21% does in Malaysia.

**Figure 6:**
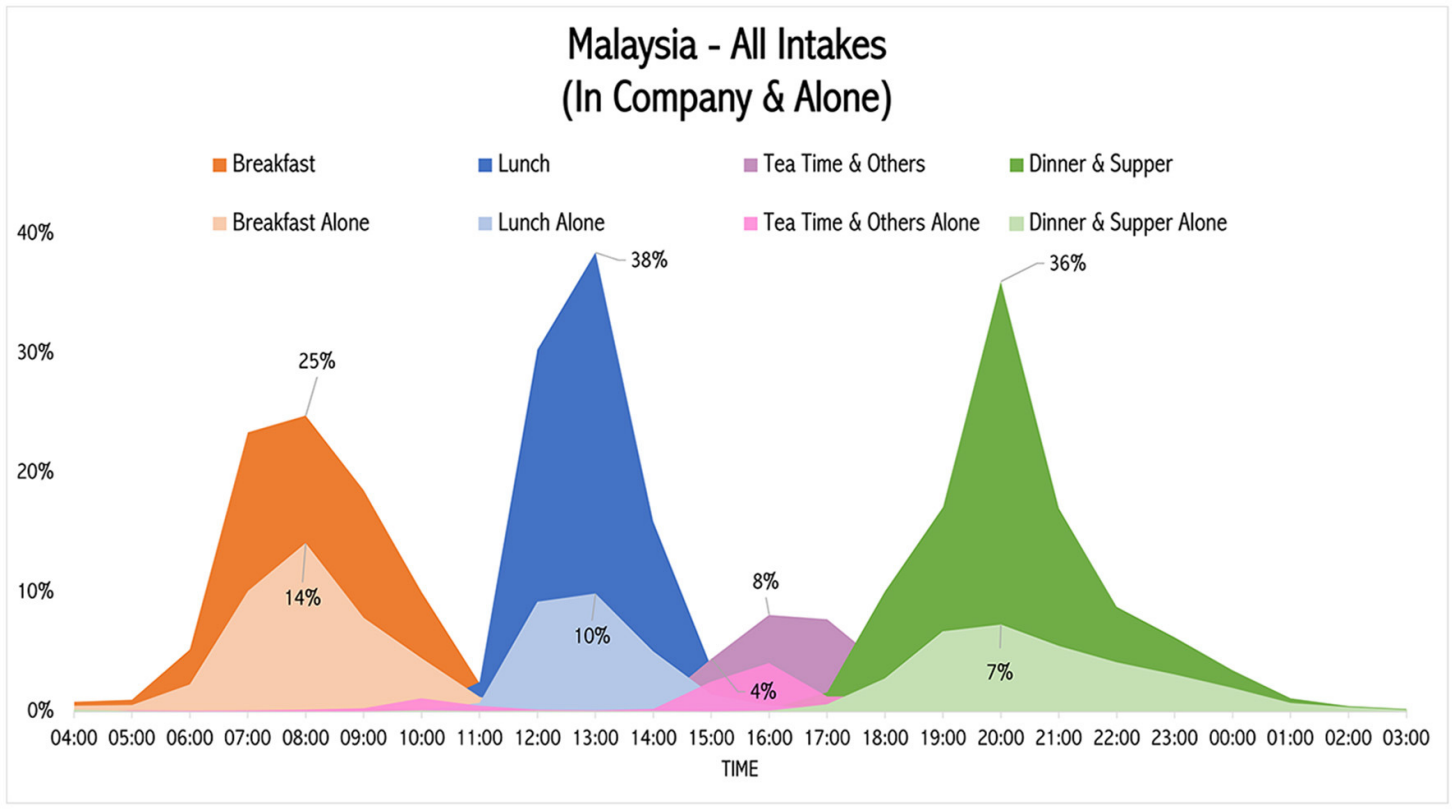
Temporal distribution of food intakes according to sociality in Malaysia.

Another analysis is the temporal distribution of the food intakes according to their sociality as displayed in Figure 6. When 38% of the Malaysian population eats lunch at 1 p.m., 10% of them are eating it alone.

## 4. Conclusion

With its open data on the repartition of preparation and incorporation of food intakes between private/domestic and public/commercial spheres, the “Eating Out” initiative provides homogenous data across five Asian countries and one occidental country. Globally, social scientists are interested in eating out from what it says about the character of contemporary societies. Studies provide details on the diverse socio-cultural and socio-historical meanings of eating out and focuses on the socio-technical and institutional arrangements (21–30). Thus, it contributes empirically to the debates on modernization (7–9) and, more specifically, on the influence of modernization on the technical and economic organization of food habits and their social and public health consequences. In view of future data collections, the data collected from early 2020 offers a baseline on food practices prior to the COVID-19 pandemic and related lockdowns. Limitations could possibly be found in the representativeness of the national samples in relation to the online collection, and the invisibility of some fluctuations in one's definition of what is home and out of home, for example, in the case of eating to another's—i.e., friend, neighbor, and family—home. Nonetheless, the analysis of the “Eating Out” dataset by researchers, public and private decision-makers, and students could benefit the food (service) industry to understand the organization of the demand of the food market, public health, as a complement to nutritional surveys in designing policies focusing on the food environment.

## Data availability statement

The datasets presented in this study can be found in online repositories. The names of the repository/repositories and accession number(s) can be found in the article/ Supplementary material.

## Ethics statement

Toluna complies with the General Data Protection Regulation 2016/679 (GDPR) and ICC/ESOMAR International Code on Market, Opinion and Social Research and Data Analytics. Written informed consent for participation was not required for this study in accordance with the national legislation and the institutional requirements.

## Author contributions

J-PP, EM, MI, NR, and PN contributed to the obtention of the funding. J-PP, EM, CL, LT, and ADu problematized and conceptualized the database. J-PP, EM, and KN designed the survey, engaged in the data collection in relation with Toluna, and drafted the manuscript. J-PP, EM, KN, and YA contributed to the construction of the constructs and data analysis. All authors contributed to manuscript revision and read and approved the submitted version.

## Footnotes

^1^This paradox could emerge from the absence of local stakeholders, particularly from the food service industry, that are economically significant enough to commission studies.

^2^Compressed or compacted modernization has been proposed by Chang (7) to refer to the civilizational condition where economic, political, social, and/or cultural changes occur in an extremely condensed manner in respect to both time and space.

^3^In sociology of food, the concept of “food day” characterises the concentration, time, and synchronization of food intakes during the day, as a result of socio-technical and institutional arrangements.

^4^To the extend where, based on her study of the foodscape from poor Jakarta (Indonesia) *kampungs*, Arciniegas (41) posits the need to revise the binary distinction between “home” and “out-of-home” eating behaviors.

^5^Frequency of meals eaten out is 1 out 5 in France, 1 out of 7 in Germany, 1 out of 4 in Italy, 1 out of 3 in UK, and 1 out 5 in Spain (40, 51).

^6^The initial questionnaire is compared with the one obtained after back-translation. Where differences are observed between the two versions, revisions along with the translators are engaged.

^7^*Toluna* employs checks to stop double participation in the data collection phase.

^8^Festive seasons are known to modify the food intake behavior. Thus, the data collection was started after New year (6 January) and then paused for Chinese New Year (23-29 January).
